# Establishment of a lysosome-related prognostic signature in breast cancer to predict immune infiltration and therapy response

**DOI:** 10.3389/fonc.2023.1325452

**Published:** 2023-12-14

**Authors:** Hairong Su, Ying Chen, Fengye Lin, Wanhua Li, Xiangyu Gu, Weijie Zeng, Dan Liu, Man Li, Shaowen Zhong, Qianjun Chen, Qubo Chen

**Affiliations:** ^1^ Second Clinical Medical College, Guangzhou University of Chinese Medicine, Guangzhou, China; ^2^ State Key Laboratory of Dampness Syndrome of Chinese Medicine, The Second Affiliated Hospital of Guangzhou University of Chinese Medicine, Guangzhou, China; ^3^ Department of Breast, Guangdong Provincial Hospital of Chinese Medicine, Guangzhou, China

**Keywords:** lysosome, breast cancer, prognosis, immune infiltration, immunotherapy, chemotherapeutic drug sensitivity

## Abstract

**Background:**

Lysosomes are instrumental in intracellular degradation and recycling, with their functional alterations holding significance in tumor growth. Nevertheless, the precise role of lysosome-related genes (LRGs) in breast cancer (BC) remains elucidated. This study aimed to establish a prognostic model for BC based on LRGs.

**Methods:**

Employing The Cancer Genome Atlas (TCGA) BC cohort as a training dataset, this study identified differentially expressed lysosome-related genes (DLRGs) through intersecting LRGs with differential expression genes (DEGs) between tumor and normal samples. A prognostic model of BC was subsequently developed using Cox regression analysis and validated within two Gene Expression Omnibus (GEO) external validation sets. Further analyses explored functional pathways, the immune microenvironment, immunotherapeutic responses, and sensitivity to chemotherapeutic drugs in different risk groups. Additionally, the mRNA and protein expression levels of genes within the risk model were examined by utilizing the Gene Expression Profiling Interactive Analysis (GEPIA) and Human Protein Atlas (HPA) databases. Clinical tissue specimens obtained from patients were gathered to validate the expression of the model genes via Real-Time Polymerase Chain Reaction (RT-PCR).

**Results:**

We developed a risk model of BC based on five specific genes (ATP6AP1, SLC7A5, EPDR1, SDC1, and PIGR). The model was validated for overall survival (OS) in two GEO validation sets (*p*=0.00034 for GSE20685 and *p*=0.0095 for GSE58812). In addition, the nomogram incorporating clinical factors showed better predictive performance. Compared to the low-risk group, the high-risk group had a higher level of certain immune cell infiltration, including regulatory T cells (Tregs) and type 2 T helper cells (Th2). The high-risk patients appeared to respond less well to general immunotherapy and chemotherapeutic drugs, according to the Tumor Immune Dysfunction and Exclusion (TIDE), Immunophenotype Score (IPS), and drug sensitivity scores. The RT-PCR results validated the expression trends of some prognostic-related genes in agreement with the previous differential expression analysis.

**Conclusion:**

Our innovative lysosome-associated signature can predict the prognosis for BC patients, offering insights for guiding subsequent immunotherapeutic and chemotherapeutic interventions. Furthermore, it has the potential to provide a scientific foundation for identifying prospective therapeutic targets.

## 1 Introduction

Over the last four decades, breast cancer (BC) incidence has escalated, emerging as a pervasive global threat to women’s health (1). The current treatments for BC have offered diverse therapeutic options, including surgery, radiation, chemotherapy, endocrine therapy, and immunotherapy (2). However, the overall prognosis for BC patients remains unfavorable, particularly for those with metastatic forms of the disease. Even with the addition of adjuvant therapy, the five-year survival rate for these individuals is still below 30% (3). Additionally, even among BC cases with seemingly identical clinical and pathological presentations, the therapeutic process proves highly complex, attributable to the biological heterogeneity of the disease and variable molecular genetic features, which may steer different therapeutic responses and prognoses (4). Therefore, developing new prognostic markers is important for enhancing treatment strategies and augmenting patient outcomes.

Lysosomes are essential components of the cellular endomembrane system, characterized by their vesicular structure enclosed by a single membrane and originating from the Golgi apparatus. The pivotal engagement of these entities in coordinating the breakdown of substances within and outside of cells via processes including endocytosis, phagocytosis, and autophagy highlights their essentiality in maintaining the balance of cellular functions and cell survival (5). Lysosomes are important players in the signaling of cellular demise since they expedite apoptosis and necrosis via lysosomal membrane permeabilization (LMP) (6, 7). Research has demonstrated the impact of lysosomal changes and malfunction on the pathogenesis of numerous illnesses, including cancer (8), neurological diseases (9), and atherosclerosis (10). The disruption of intracellular homeostasis caused by lysosomal dysregulation is a key factor in the proliferation and survival of neoplasms (11). Furthermore, the process of lysosomal translocation and abnormal secretion augments the invasiveness of cancer cells and their capacity to metastasize (12).

Multiple studies have emphasized the significant benefits linked to lysosomes in tumor diagnosis, prognosis, and therapeutic approach. An increase in cathepsin levels within lysosomes correlates with the progression of tumors, indicating an unfavorable prognosis for tumor development. The identification of specific cathepsins presents potential opportunities in both prognostication and therapeutic intervention (13, 14). Similarly, genes related to lysosomes have demonstrated significance as indicators for cancer diagnosis and prognosis. The upregulation of TFEB, a critical regulator in the autophagy-lysosomal pathway, has been connected with poor cancer prognosis. The experimental findings indicated that a reduction in TFEB expression was linked to a decline in the ability of cancer cells to resist radiation, implying that the lysosome plays a noteworthy role in autophagy-mediated radioresistance (15). In addition, the targeting of the lysosomal system is regarded as a possible therapeutic approach. During malignant transformation, tumor cells are vulnerable to LMP. Based on this shortcoming, Wang et al. created mainly targeted medications to induce lysosomal degradation and trigger lysosome-associated liver cancer cell killing (16). These researches suggested that lysosome-related genes (LRGs) could potentially serve as new markers for tumors, providing guidance for treatment decisions and improving the accuracy of prognostic assessments. Recent studies confirmed that lysosome-associated signatures exhibited potential capacities for predicting prognosis in malignancies, including gastric and lung cancer (17, 18). Nevertheless, there is a lack of studies on predictive BC models based on LRGs. This emphasizes the necessity of conducting studies on LRGs within cohorts of BC patients.

In our study, we analyzed the differentially expressed genes (DEGs) among BC and normal samples using public databases to establish a gene signature of five differentially expressed lysosome-related genes (DLRGs). The validation of the signature was conducted in two separate cohorts, with BC patients being classified into two distinct groups according to their respective risk levels. Further comparative analyses of these groups included pathway enrichment, characterization of the tumor microenvironment, evaluation of immunotherapy response, and assessment of drug sensitivity. In summary, our novel BC prognostic model based on the five DLRGs suggested new therapeutic recommendations for clinical practice.

## 2 Materials and methods

### 2.1 Sources and processing of datasets

**Figure 1:**
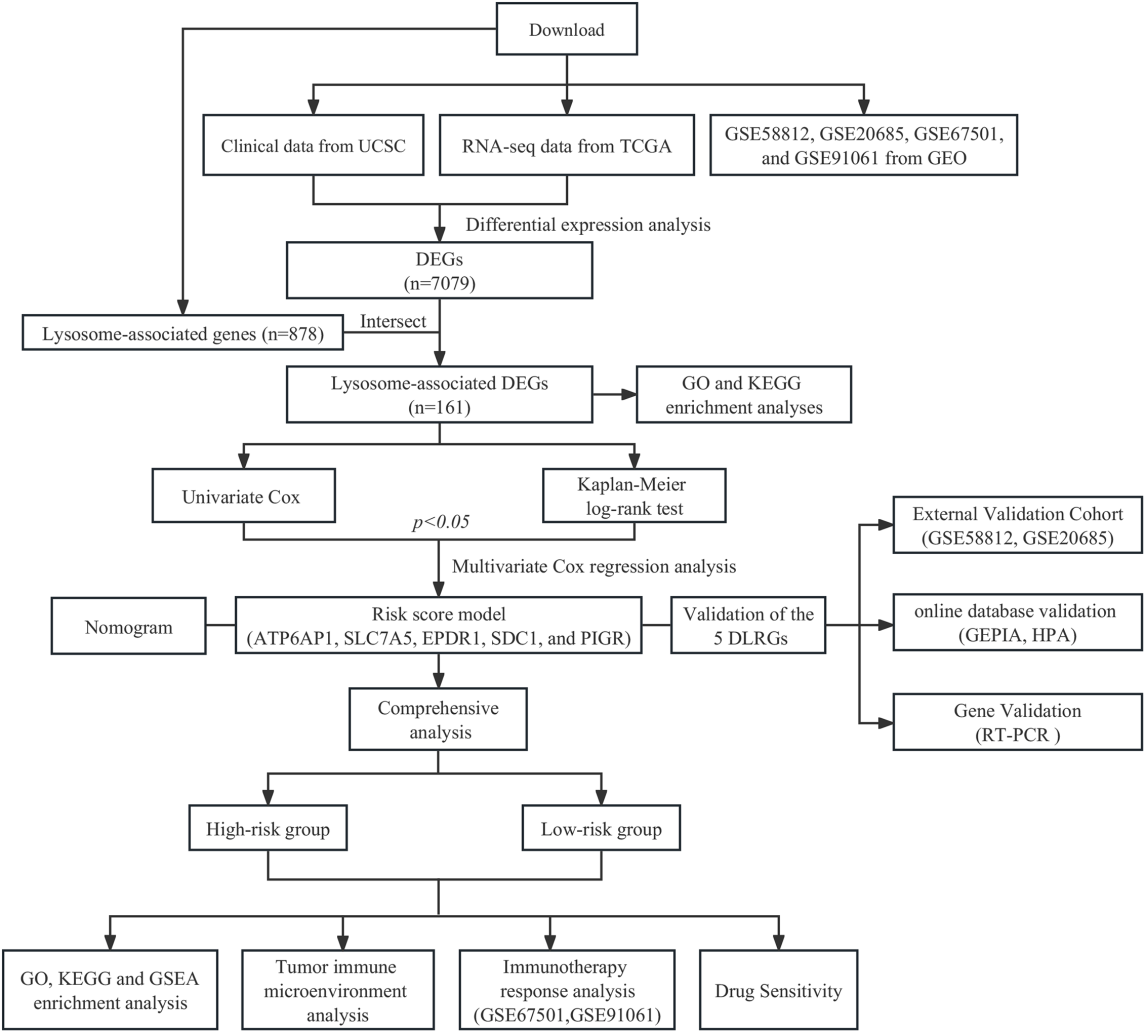
Workflow of the study.

The clinical data and gene expression profiles for BC samples were obtained from The Cancer Genome Atlas (TCGA; https://portal.gdc.cancer.gov/). After excluding male patients and individuals with survival durations below 30 days, the study retained 113 normal and 1,049 BC samples for subsequent examination. Differential expression analysis was conducted using count data obtained from the TCGA cohort, while TPM data were employed for other evaluations. RNA sequencing data along with clinical metadata for two validation sets (GSE20685 and GSE58812) were sourced from the Gene Expression Omnibus (GEO; https://www.ncbi.nlm.nih.gov/gds/), including 107 and 327 BC samples, respectively. The clinical attributes of each cohort are presented in **Supplementary Table 1**. 878 LRGs were extracted from the Gene Ontology (GO) database (http://geneontology.org/), detailed in **Supplementary Table 2**. Additionally, two cohorts of immunotherapy (GSE67501 and GSE91061) downloaded from GEO were included in our study (19, 20). **Supplementary Table 3** presents the information regarding the response to immunotherapy. The workflow of this study is shown in **Figure 1**.

### 2.2 Identification of differential LRGs and enrichment analysis in BC

The identification of DEGs in the TCGA dataset, between BC and normal samples, was performed utilizing the “limma” package. A significance criterion was established at an adjusted *p*-value of less than 0.05 coupled with an absolute logFC value exceeding 1. By intersecting DEGs with LRGs, DLRGs were delineated. Subsequent visual representation of the results employed an assortment of R packages, including “VennDiagram” for a Venn diagram, “EnhancedVolcano” for a volcano plot, and “ComplexHeatmap” for a heatmap. The potential pathways linked to DLRGs were explored in GO and the Kyoto Encyclopedia of Genes and Genomes (KEGG). The “clusterProfiler” package was employed in the R programming language with a selection criterion of an adjusted *p*-value below 0.05.

### 2.3 Establishment of LRGs signature

An examination of univariate Cox regression was executed on DLRGs (*p*< 0.05). This was followed by a Kaplan-Meier (KM) log-rank test to further identify the predictive significance (*p*< 0.05) of DLRGs. The pinpointed genes were subsequently analyzed using stepwise multivariate Cox regression to hone the predictive model. Ultimately, a novel gene signature linked with lysosomes, which can be used for predicting prognosis, was developed through this multivariate analysis.

A risk score founded on LRGs was derived with the formula: Riskscore = $\sum^{}\text{gene expression }\times \text{gene Cox coefficient}$ . The median was designated as the cut-off value according to the signature score of the LRGs.

### 2.4 Evaluation of the prognostic value of the LRGs signature

In order to assess the prognostic potential of the LRGs signature, we computed scores for BC patients from TCGA, GSE20685, and GSE58812 cohorts following the risk score formula. Based on predefined thresholds, patients were classified into low-risk or high-risk categories. KM survival analyses applying the “survminer” package were conducted to examine the differences in overall survival (OS) for the two different risk groups. The study employed the time-dependent receiver operating characteristic (timeROC) methodology to evaluate the prediction efficacy of the model. To evaluate the independent prognostic value of the LRGs signature, we conducted regression analyses comparing it with clinicopathological features. Clinical factors deemed significant were integrated into a nomogram and visualized by the “regplot” package. The predictive power of the model was evaluated with the Concordance index (C-index), which serves as a metric for assessing the model’s accuracy in predicting patient events correctly. Calibration plots illustrated the congruence between predicted and observed survival outcomes. For a comprehensive assessment of survival prognosis, the “timeROC” method provided evaluations at the 3-, 5-, and 10-year marks. The clinical applicability of our nomogram model was further elucidated by the Decision Curve Analysis (DCA). To enhance the credibility of our findings, we validated the prognostic nomograms in the GSE20685 and GSE58812 datasets.

### 2.5 Pathway analysis of the LRGs signature

We identified DEGs in both high- and low-risk categories within the TCGA cohort. To elucidate the biological importance of these genes, we conducted GO analysis and utilized the KEGG for pathway enrichment. Additionally, the Gene Set Enrichment Analysis (GSEA) carried out using the “clusterProfiler” package to identify the predominant pathways in each risk group. Results obtained after 1,000 permutations with an FDR significance level of 0.25 and a *p*-value less than 0.05.

### 2.6 Exploration of tumor immune microenvironment

The single-sample gene set enrichment analysis (ssGSEA) was used to determine the infiltration levels of 28 immune cell types (21). To examine the varying degrees of immune infiltration within risk groups, the “estimate” package was utilized to compute stromal, immune, and ESTIMATE scores for each tumor sample (22). Spearman correlation analysis was utilized to evaluate the relationship between the expression of 5 DLRGs and immune cell abundance. Additionally, we investigated the relationship between the risk score and the three scores derived from the ESTIMATE method. Subsequently, we employed the CIBERSORT algorithm and ssGSEA methodologies to investigate the disparities in the immune cell composition among the various groups (23). Additionally, we scrutinized the expression of human leucocyte antigen (HLA) genes, encompassing both MHC class I and MHC class II molecules.

### 2.7 Prediction of the therapeutic response to immune checkpoint inhibitors

The Tumor Immune Dysfunction and Exclusion (TIDE; http://tide.dfci.harvard.edu/) platform offers significant contributions to our understanding of the tumor microenvironment, presenting a streamlined approach for predicting immune checkpoint inhibitors (ICIs) response (24, 25). By inputting the transcriptomic profiles of 1,049 BC samples, we ascertained their possible responsiveness to ICIs. The Immunophenotypic Score (IPS), with a scale from 0-10, gauges the immunogenicity of a patient and provides a predictive measure of their probability to respond to ICIs, including PD1/PDL1/PDL2 and CTLA4 blockers (26). Utilizing The Cancer Immunome Atlas (TCIA; https://tcia.at/), we assessed the IPS for 1,049 BC samples, highlighting differences in immunotherapeutic potential across risk groups. The immune response rates of various risk groups were used in two immunotherapy cohorts (anti-PD-1 in the GSE67501 cohort, anti-CTLA4 and anti-PD-1 in the GSE91061 cohort), to validate the predictive capability of the developed signatures in determining the efficacy of immunotherapy. Moreover, we adopted a Pearson correlation to find links between immune checkpoints (PD-L1, PD1, and CTLA4) and specific genes found in our prognostic model.

### 2.8 Drug sensitivity analysis

Drawing from the Genomics of Drug Sensitivity in Cancer (GDSC) database (http://www.cancerrxgene.org/), we employed the “oncoPredict” package to ascertain drug sensitivity scores for cohorts categorized as high- and low-risk groups (27). A lower score signifies heightened sensitivity to drugs. To further delineate the association between the risk score and medicine sensitivity, we plotted a scatterplot underpinned by Spearman correlation analysis.

### 2.9 Verification of the LRGs signature in databases

The expression of the LRGs signature was verified by online public databases. Through GEPIA (http://gepia.cancer-pku.cn/index.html), we examined the mRNA expression levels of five DLRGs in both BC and normal tissues, referencing data from TCGA and genotype-tissue expression (GTEx). In order to further examine protein expression, we consulted the Human Protein Atlas (HPA) database (https://www.proteinatlas.org/), where we analyzed immunohistochemistry (IHC) images and their corresponding staining intensities. The HPA categorizes expression as high, medium, low, or undetected, depending on staining percentages and intensities. We also collated the staining data for these DLRGs across both BC and normal tissues from HPA.

### 2.10 Clinical samples

For this study, we exclusively selected patients diagnosed with BC who had not received any prior radiotherapy or chemotherapy treatments before their surgical procedures. From August to September 2023, a total of 13 patients who were scheduled for BC surgery at the Guangdong Provincial Hospital of Chinese Medicine were included in this study. Our research adhered to the Declaration of Helsinki (revised 2013) and obtained approval from the hospital’s Ethics Committee (BF2019-120-03). All patients provided informed consent.

### 2.11 Real-time polymerase chain reaction

Total RNA from BC and adjacent non-tumor tissues was extracted using TRizol (Thermo, USA). The integrity and quality of RNA were detected on a NanoDrop 2000 spectrophotometer (Thermo, USA). The reverse transcription of RNA to cDNA was performed by the Evo M-MLV Reverse Transcription Premix Kit (Accurate Biotechnology, Changsha, China). The LightCycler II 480 instrument (Roche, Switzerland) was used to perform duplicate RT-PCR studies. The SYBR Green Pro Taq HS qPCR kit (Accurate Biotechnology, Changsha, China) was employed for this purpose. The mRNA expression levels of the gene were normalized by β-actin and quantified with the 2^-ΔΔCT^ technique. The primer sequences are available in **Supplementary Table 4**.

### 2.12 Statistical analysis

Statistical evaluations were executed utilizing R software (version 4.3.0) and GraphPad Prism (version 9.5.0). The Wilcoxon signed-rank test was adopted to evaluate the presence of significant differences between the groups. For paired tissue samples, we employed either a paired-samples t-test or the paired-samples nonparametric Wilcoxon signed-rank test. All analyses were two-tailed, and statistical significance was determined with p-values less than 0.05. The signs for significance were as follows: *p ≤ 0.05, **p ≤ 0.01, and ***p ≤ 0.001.

## 3 Results

### 3.1 Identification of 161 DLRGs in BC patients

**Figure 2:**
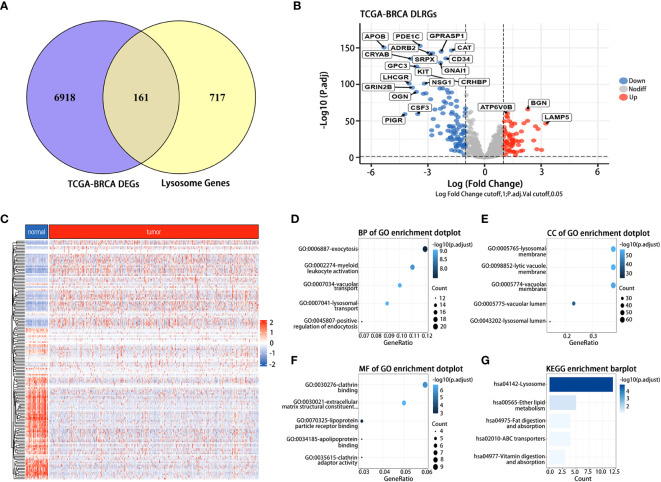
Identification and functional analysis of DLRGs in BC. **(A)** Venn diagram of DLRGs. **(B)** Volcano plot of DLRGs. **(C)** Heatmap of 161 DLRGs in tumor and normal samples. **(D–G)** Functional Analysis of DLRGs in GO and KEGG pathways.

In the TCGA dataset, we discerned 7,079 DEGs between BC tumor and normal samples. 161 DLRGs were identified based on Venn analysis (**Figure 2A**). Of these, 63 showed upregulation, while 98 exhibited downregulation (**Figures 2B, C** and **Supplementary Table 5**). The enrichment analysis revealed notable associations with pathways involved in lysosomal transport, vacuolar movement, exocytosis, and positive regulation of endocytosis (**Figures 2D–F**). The KEGG analysis emphasized their involvement in the lysosome and ether lipid metabolism pathways (**Figure 2G** and **Supplementary Table 6**).

### 3.2 Construction and validation of the LRGs signature

**Figure 3:**
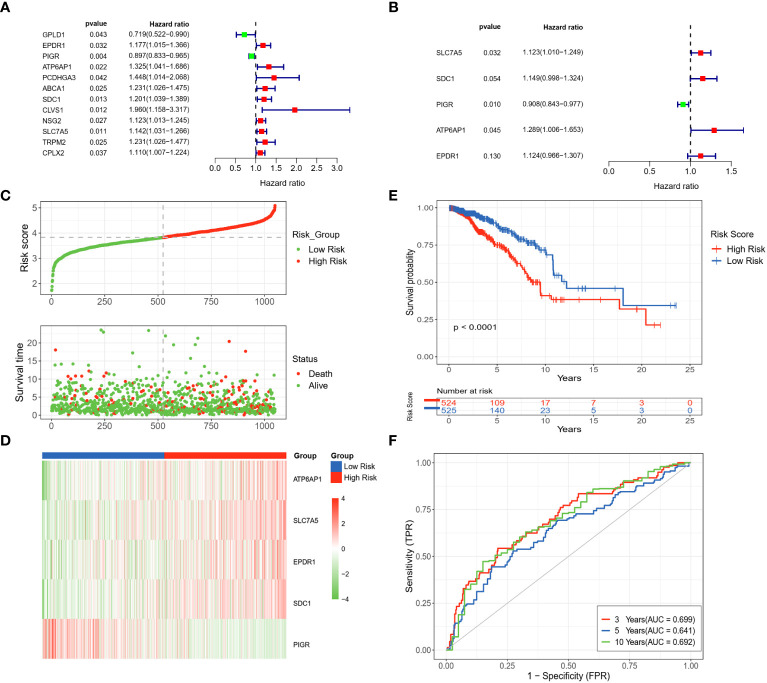
Development and assessment of a LRGs signature in the TCGA dataset. **(A)** Forest plot of the 12 DLRGs identified via univariate Cox regression analysis. **(B)** Forest plot of the 5 DLRGs identified via multivariate Cox regression analysis. **(C)** The risk curve and scatter plot of high- and low-risk groups in the TCGA cohort. **(D)** The heatmap of 5 DLRGs expressions for high- and low-risk groups. **(E)** Comparison of the OS in the high- and low-risk groups using Kaplan-Meier survival curves. **(F)** Time-dependent ROC curves.

From univariate Cox regression analysis, ten DLRGs were identified as potential risk factors associated with unfavorable BC outcomes (HR > 1, *p<* 0.05) (**Figure 3A**). In contrast, GPLD1 and PIGR demonstrated protective effects (HR< 1, *p<* 0.05). KM log-rank tests further refined prognostically relevant lysosome-associated genes (*p<* 0.05) (**Supplementary Table 7**). The results of stepwise multivariate Cox regression finalized five genes (SLC7A5, SDC1, PIGR, ATP6AP1, and EPDR1) for prognostic model development (**Figure 3B**). The derived risk score is: Riskscore = SLC7A5 × (0.1161780) + SDC1 × (0.1392586) + PIGR × (-0.0966812) + ATP6AP1 × (0.2540440) + EPDR1 × (0.1168528). Based on the median value of their risk score, patients were categorized into low- and high-risk groups. It was observed that the high-risk group exhibited an increased expression of poor prognosis genes (**Figures 3C, D**). The KM survival analysis affirmed that OS was markedly diminished in high-risk patients compared to those deemed low-risk (*p<* 0.0001) (**Figure 3E**). Moreover, the timeROC curve elucidated the AUC values for OS predictions at various time points, revealing AUCs of 0.699, 0.641, and 0.692 for the 3-year, 5-year, and 10-year forecasts, respectively (**Figure 3F**). Our findings suggested that the computed risk scores had high accuracy in prognosticating OS outcomes.

**Figure 4:**
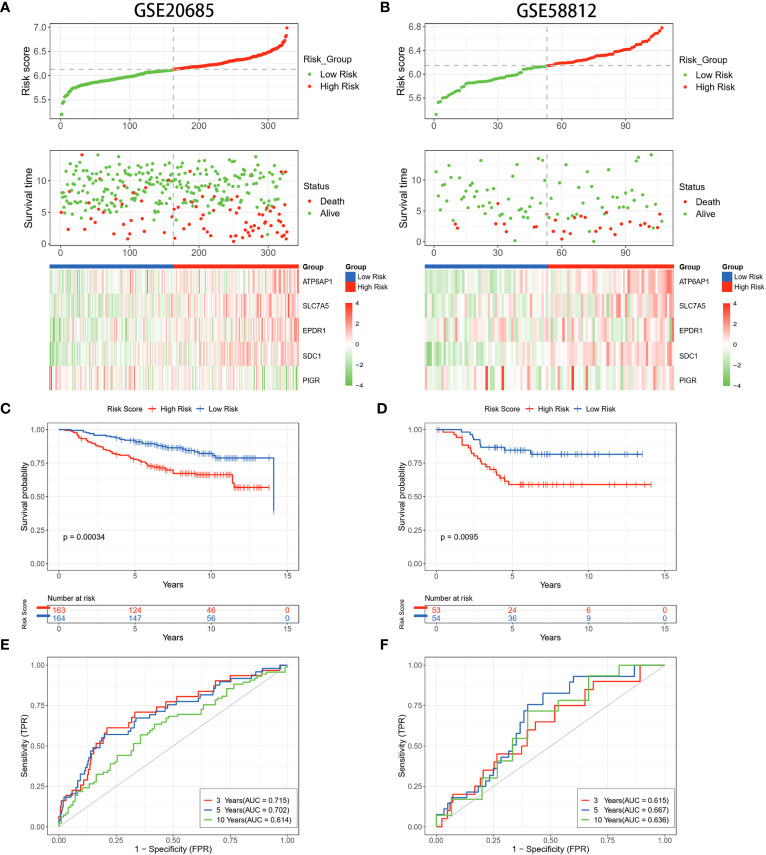
Validation of the LRGs signature in the GEO datasets. **(A, B)** The risk curves, scatter plots, and 5 DLRGs expression heatmaps of high- and low-risk groups in the GSE20685 and GSE58812 cohorts. **(C, D)** Comparison of the OS in the high- and low-risk groups using Kaplan-Meier survival curves in two GEO cohorts. **(E, F)** Time-dependent ROC curves of the GEO cohorts.

To validate the stability of risk scores for OS prediction, datasets GSE20685 and GSE58812 were utilized as external validation sets to repeat the above tests. The findings pertaining to the distribution of risk scores, survival outcomes, and expression profiles of BC patients were in alignment with the results acquired from the TCGA database (**Figures 4A, B**). The predictive capability of the risk scores remained consistent in predicting the prognosis of BC patients. Specifically, patients identified as high-risk demonstrated notably shorter OS compared to those in the low-risk group. (*p* = 0.00034, *p* = 0.0095) (**Figures 4C, D**). The timeROC curves demonstrated the AUC values of 0.715 and 0.615 (3 years), 0.702 and 0.667 (5 years), and 0.614 and 0.636 (10 years) for GSE20685 and GSE58812, respectively (**Figures 4E, F**). These results collectively underscored the reliability of the LRGs signature in forecasting the prognosis of BC.

### 3.3 Independent prognostic value of the LRGs signature

**Figure 5:**
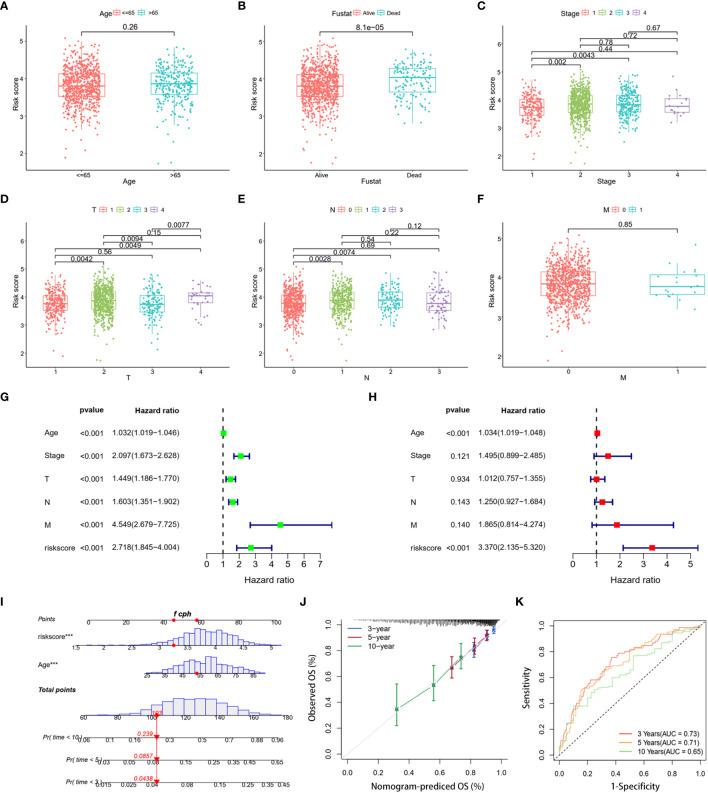
Clinical correlation analysis and creation of a nomogram. Scatter plots of age **(A)**, survival status **(B)**, stage **(C)**, and stages T **(D)**, N **(E)**, and M **(F)** for two risk groups. **(G, H)** The risk score and clinical variables in the TCGA cohort were analyzed using univariate and multivariate Cox regression. **(I)** Nomogram using clinical factors and risk score to estimate BC OS for 3, 5, and 10 years. **(J)** Calibration curves for 3, 5, and 10 years. **(K)** The 3-, 5-, and 10-year OS ROC curves for the nomogram. ***p< 0.001.

To refine the risk model, we combined clinical features to develop a prognostic nomogram for BC. The heatmap elucidated the association between clinicopathologic features and the expression trends for five DLRGs (**Supplementary Figure 1**). The scatterplot analysis revealed statistically significant differences between risk scores and survival status, stage, T stage, and N stage (**Figures 5B–E**). However, there were no substantial disparities in risk scores with respect to age (**Figure 5A**) or M stage (**Figure 5F**). In addition, univariate and multivariate Cox regression analyses assessed the potential independence of clinicopathologic characteristics and risk scores as prognostic factors in BC (**Figures 5G, H**). The results of our study indicated that both age and risk score could act as independent prognostic indicators for patients with BC. Notably, the risk score demonstrated pronounced influence on prognosis. This emphasized the independent prognostic capacity of our risk score. Upon integrating age and the risk score, our nomogram attained a C-index of 0.691, indicating robust predictive precision (**Figure 5I**). Calibration curves confirmed the alignment between predicted and actual outcomes (**Figure 5J**). Evaluations using the TCGA training dataset revealed AUC values of 0.73, 0.71, and 0.65 at 3-, 5-, and 10- year, respectively (**Figure 5K**). Concurrently, the GSE20685 and GSE58812 datasets manifested similar AUC metrics, and DCA further validated the substantial net clinical benefit conferred by our nomogram (**Supplementary Figure 2**).

### 3.4 Functional evaluation of the LRGs signature

**Figure 6:**
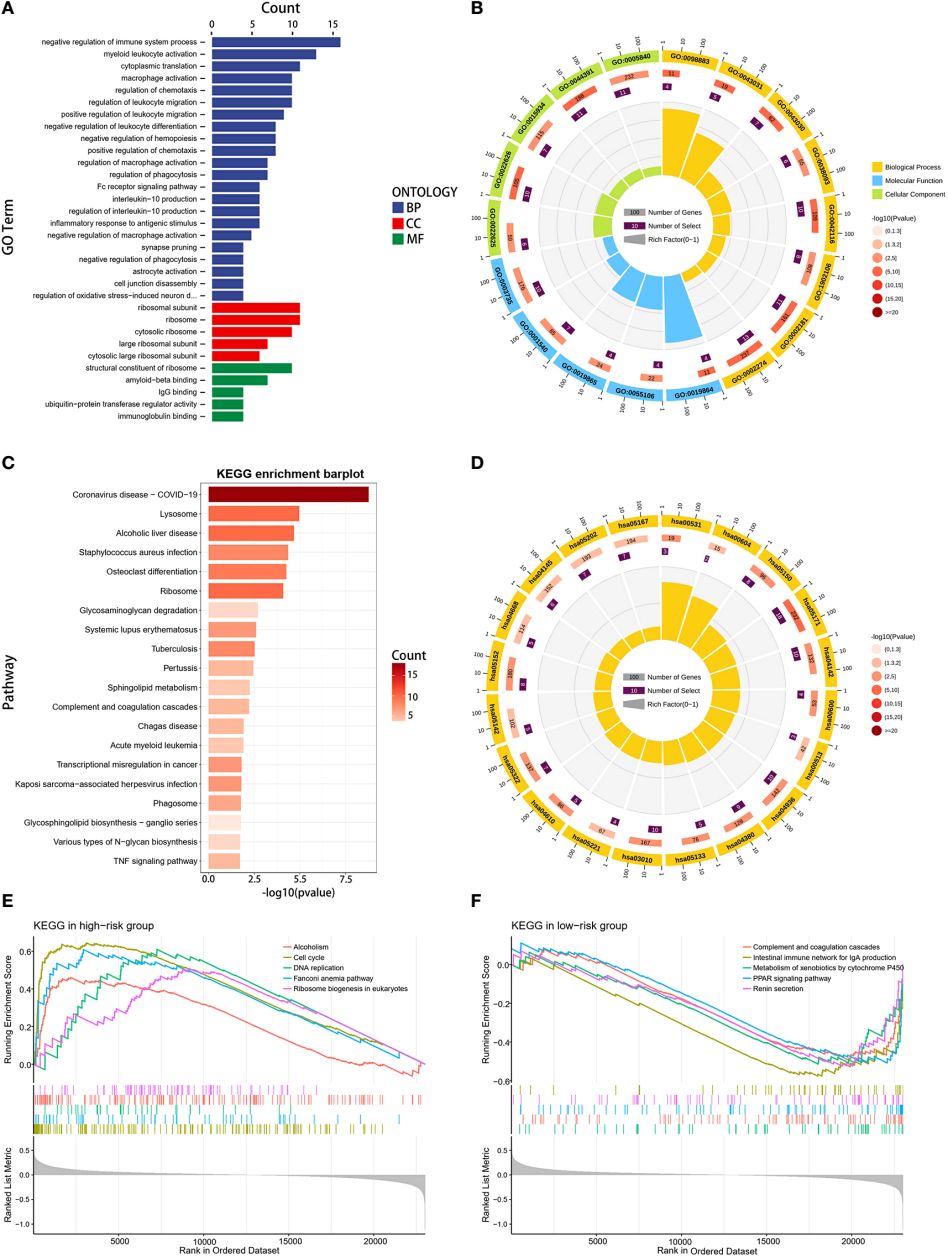
Functional enrichment analysis. **(A, B)** GO enrichment analysis for DEGs between high- and low-risk groups. **(C, D)** KEGG enrichment analysis for DEGs between the two risk groups. **(E, F)** GSEA enrichment analysis in the two risk groups.

To gain deeper insights into the biological functions of the two established risk categories, comprehensive analyses were conducted using GO, KEGG, and GSEA analyses. The GO analysis underscored a significant enrichment in immune-related biological activities, particularly in aspects such as the negative regulation of immune system processes, activation of myeloid leukocytes, macrophage activation, and migration of leukocytes (**Figures 6A, B**). The KEGG analysis identified that the genes exhibiting differential expression were primarily linked to coronavirus disease (COVID-19), lysosome function, and alcoholic liver disease (**Figures 6C, D**). Furthermore, GSEA reinforced the finding that pathways linked to the cell cycle and DNA replication were markedly prevalent in the group with higher risk, suggesting a pronounced proliferative tendency in these patients (**Figure 6E**). In contrast, the group with lower risk demonstrated a pronounced involvement in immune-related pathways, including activation of PPAR signaling, complement cascade reactions, IgA intestinal immune network, and enhanced metabolic processes such as exogenous metabolism and renin secretion (**Figure 6F**). The results of our study revealed marked differences in immunological activity, cellular proliferation, and metabolic profiles between the two risk groups. These variations could potentially elucidate the differential survival rates observed in our study population.

### 3.5 Different tumor immune microenvironment of two risk groups

**Figure 7:**
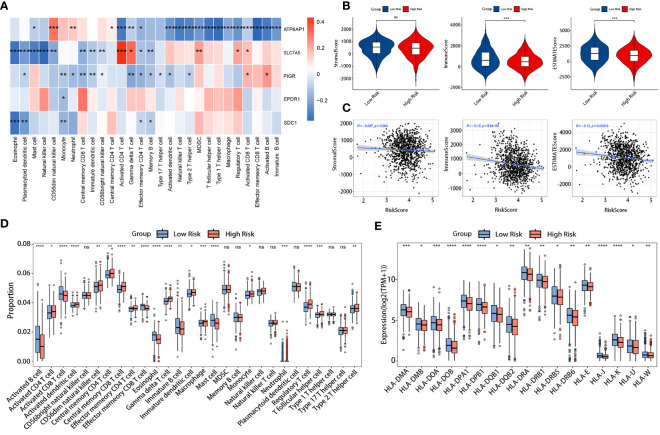
Comparison of TIM in the TCGA cohort. **(A)** Correlation heatmap of the 5 DLRGs and immune cells. **(B)** Differences in the three scores of ESTIMATE results between the two risk groups. **(C)** The association between the risk scores and the three scores of ESTIMATE results. **(D)** Differences in 28 immune cells between the two risk groups. **(E)** The expression levels of HLA genes between two risk groups. *p< 0.05, **p< 0.01, ***p< 0.001, ****p< 0.0001. ns, no significance.

The tumor immune microenvironment (TIM) acted as a significant indicator of the biological behavior shown by tumors. We investigated the relationship between the LRGs signature and the infiltration levels of 28 different types of immune cells. The results indicated that five DLRGs showed a negative correlation with the majority of the immune cells. (**Figure 7A**). Furthermore, some of these genes were found to be associated with the infiltration of effector memeory CD8 T cells and activated CD4 T cells. These findings implied a potential association between our signature and the TIM. The low-risk group manifested superior immune and ESTIMATE scores, indicating a higher presence of immune cells surrounding their tumors, whereas stromal scores did not significantly differ between the two groups (**Figure 7B**). The risk scores and the three ESTIMATE-derived scores showed a negative connection (**Figure 7C**). There was a statistically significant difference in the abundance of distinct immune cell types between the two risk categories as determined by ssGSEA analysis (**Figure 7D**). Fourteen distinct immune cell types exhibited increased infiltration in the high-risk group, including regulatory T cells (Tregs) and type 2 T helper cells (Th2). In contrast, there was a higher abundance of lymphocytes such as activated B cells and CD8 T cells in the low-risk group. **Supplementary Figure 3** displayed the comparative abundance of 22 distinct immune cell subtypes in the high- and low-risk groups. Moreover, the expression of HLA genes was significantly upregulated in the low-risk group (**Figure 7E**). This implied that low-risk patients possessed a greater antigen-presenting capacity, which may lead to an increased efficacy of immunotherapy. Collectively, our results demonstrated differences in the immune microenvironments between patients classified as low-risk and high-risk.

### 3.6 Prediction of response to immunotherapy

**Figure 8:**
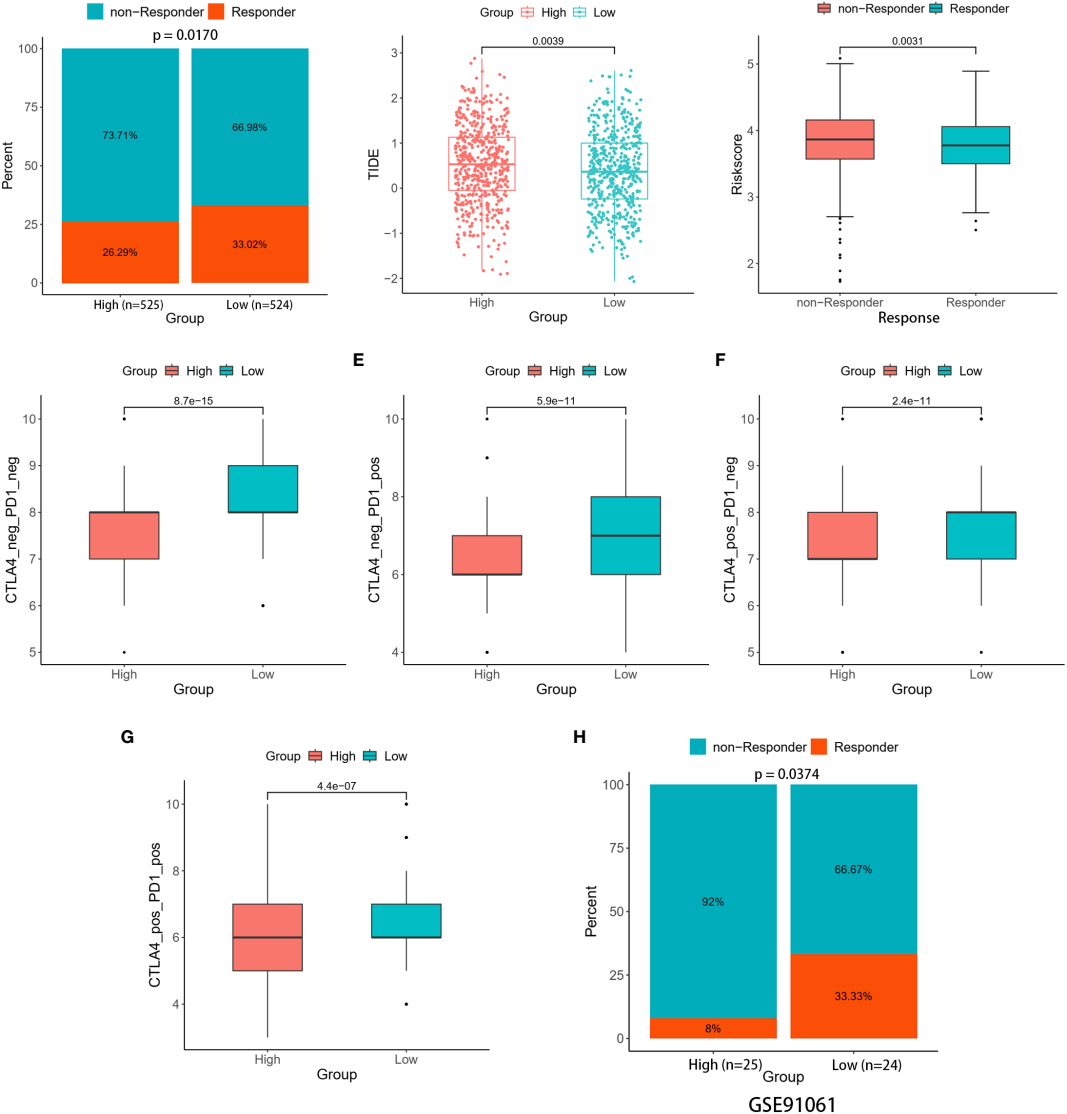
Immune therapy response between the two risk groups. **(A)** Percentage of responders and non-responders in the two risk groups of the TCGA cohort. **(B)** Differences in TIDE score between the two risk groups. **(C)** Differences in risk score among the responders and non-responders. **(D–G)** Differences in Four IPS values between the two risk groups. **(H)** Percentage of responders and non-responders in the two risk groups of the GSE91061 cohort.

Making use of TIDE, we investigated the immunotherapeutic responses in the high- and low-risk patients. The findings from the TIDE study demonstrated that low-risk patients exhibited a more favorable response to ICIs than individuals in the high-risk group (33.02% versus 26.29%, *p* = 0.0170) (**Figure 8A**). Meanwhile, reduced TIDE scores observed in the low-risk group suggested a more favorable response to immunotherapy (**Figure 8B**). Furthermore, our study showed that responders had risk scores significantly lower than non-responders (**Figure 8C**). These observations implied a potential link between the risk scores and the efficacy of immunotherapy treatments. The vulnerability of individuals to ICIs was further evaluated by the IPS. The results showed that low-risk patients exhibited higher values across all CTLA4 and PD-1 stratifications compared to their high-risk counterparts, suggesting that the likelihood of a positive response to ICIs was greater as opposed to the high-risk group (**Figures 8D–G**).

The ability of the signature to predict responses to immunotherapy was confirmed through validation in two distinct cohorts (GSE67501 and GSE91061). In the GSE67501 cohort, although not statistically significant, a higher percentage of low-risk patients exhibited a response to anti-PD-1 treatment (low-risk vs. high-risk, 40.00% vs. 33.33%, Fisher’s exact test, *p* > 0.05) (**Supplementary Figure 4**). In the GSE91061 cohort, the low-risk group exhibited a significantly higher rate of immune response (low-risk vs. high-risk, 33.33% vs. 8.00%, Fisher’s exact test, *p* = 0.0374) (**Figure 8H**). Consequently, the two distinct risk groups, based on the LRGs signature, exhibited disparate responses to immunotherapy. Notably, individuals classified as low-risk demonstrated heightened sensitivity to immunotherapy, resulting in more favorable clinical outcomes. Further investigation into this phenomenon uncovered a relationship between the expression levels of five DLRGs and three pivotal immune checkpoint markers (PD-L1 (CD274), CTLA4, and PD1 (PDCD1)) (**Supplementary Figure 4**). Specifically, ATP6AP1 demonstrated a negative correlation with these immune checkpoint genes, whereas SLC7A5 and PIGR showed positive correlations.

### 3.7 Analysis of the correlation between risk score and drug sensitivity

**Figure 9:**
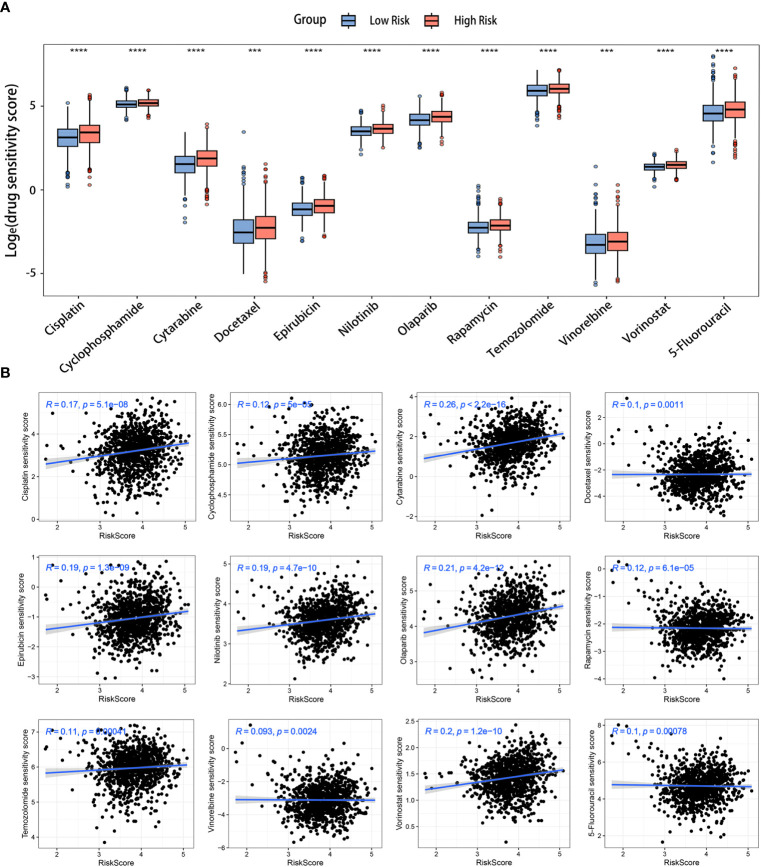
Prediction of chemotherapy drugs for BC in the two risk groups. **(A)** Differences in chemotherapeutic drug sensitivity scores between the two risk groups. **(B)** Scatter diagrams displaying the relationships between the risk scores and chemotherapeutic drug sensitivity scores. ***p< 0.001, ****p< 0.0001.

The drug sensitivity analysis was conducted to evaluate the reliability of our signature in predicting the response to chemotherapy among BC patients. A lower drug sensitivity score corresponded to increased sensitivity to the treatment. The sensitivity of various chemotherapeutic drugs commonly employed in clinical practice, including cyclophosphamide (an alkylating agent), 5-fluorouracil (a thymidylate synthase inhibitor), epirubicin (a topoisomerase inhibitor), and docetaxel (a microtubule inhibitor), among others, exhibited greater sensitivity in the low-risk group (**Figure 9A**). Additionally, the outcomes of the correlation analysis indicated a positive association between medicine sensitivity scores and risk scores (**Figure 9B**). Certain chemotherapy medications, specifically cytarabine, olaparib, and vorinostat, were found to be highly connected with risk scores. These critical observations suggested a potential disparity in medication resistance between high- and low-risk BC patients. The risk scores based on the LRGs signature might be valuable for selecting effective clinical chemotherapeutic agents.

### 3.8 Validation of the expression of the LRGs signature

**Figure 10:**
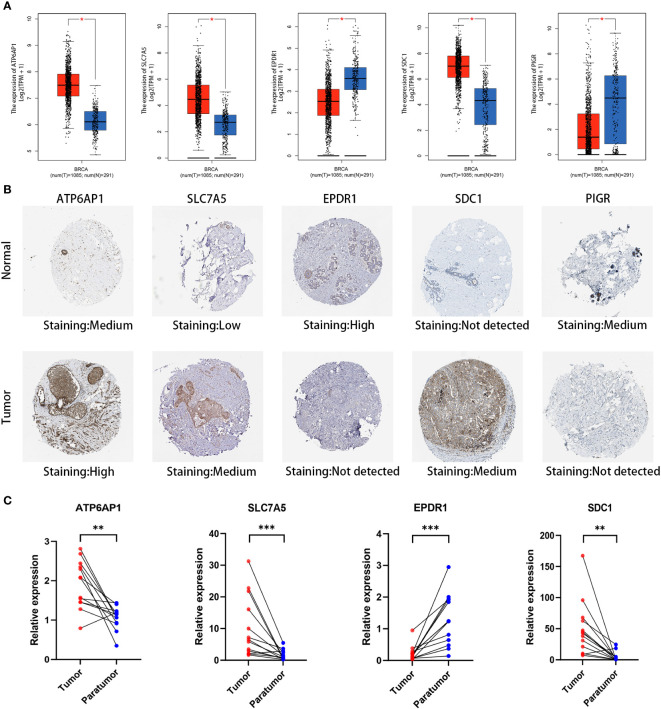
Validation of five DLRGs in the prognostic model. **(A)** Expression comparisons of the 5 DLRGs between tumor and normal tissues using GEPIA. **(B)** Immunohistochemical staining for 5 DLRGs between tumor and normal tissues using HPA. **(C)** The mRNA relative expression of ATP6AP1, SLC7A5, EPDR1, and SDC1 in 13 paired tumor tissues. N, normal tissue; T, tumor tissue. *p< 0.05, **p< 0.01, ***p< 0.001.

We performed an in-depth examination of the mRNA and protein expression profiles of the five DLRGs in BC in order to validate them. The GEPIA database analysis revealed a significant elevation in mRNA expression levels of ATP6AP1, SLC7A5, and SDC1 within BC tissues. Conversely, EPDR1 and PIGR exhibited markedly decreased mRNA expression (**Figure 10A**). Corroborating these findings, IHC images from the HPA database revealed the upregulation of ATP6AP1, SLC7A5, and SDC1 proteins in BC tissues, while EPDR1 and PIGR proteins displayed diminished expression (**Figure 10B**). We summarized the IHC staining features of the five DLRGs, which provided results that were consistent with the previously described data (**Supplementary Figure 5**). Furthermore, the findings of IHC investigations in BC conducted by other researchers have been identified for SLC7A5 and SDC1, providing additional support for the results documented in the HPA database (28, 29).

To enhance our insights into the prognostic validity of the five-gene signature, we examined the expression of ATP6AP1, SLC7A5, EPDR1, SDC1, and PIGR in 13 paired BC samples, comparing them with their adjacent non-tumor tissues through RT-PCR. Remarkably, ATP6AP1, SLC7A5, and SDC1 displayed heightened expression in BC, while EPDR1 evidenced a significant down-regulation (**Figure 10C**). Given the limited sample set, the expression of PIGR showed no marked contrast between BC and its corresponding non-tumor tissues. A broader set of tissue samples is necessary to validate these expression trends.

## 4 Discussion

Lysosomes, membrane-bound organelles recognized for their digestive functions, are essential in waste disposal, signaling, and energy production in cells (30). Recent studies underscored the profound influence of lysosomal functional integrity and distribution on tumorigenesis and tumor progression. Beyond impacting the proliferation, movement, and infiltration of cancerous cells (26), lysosomes contribute to resistance against chemical therapies, modify the tumor’s surrounding environment, and direct the orientation of associated immune cells within tumors, specifically macrophages (31). In BC research, key studies have linked the disease to four autophagy-lysosomal pathway genes: TMEM175, SCARB2, ATG16L2, and TMEM63A (32). Importantly, IITZ-01, recognized as a lysosomal autophagy inhibitor, emerges as a promising therapeutic option, particularly for triple-negative BC (33). Driven by these insights, we investigated the association between lysosome-associated genes and the outcomes for BC patients, as well as immune infiltration, immunotherapy, and drug susceptibility. Through this research, we aimed to propel advancements in BC treatment and enhance patient prognosis.

In our study, we focused on multiple LRGs, which led to the successful construction of a novel lysosome-related prognostic model for BC. Employing risk evaluations formulated through our model, we divided BC patients from three separate cohorts into classifications of high and low risk. Our analysis revealed a substantial variation in OS among these groups, with those in the high-risk group demonstrating a markedly diminished prognosis. We determined that the risk score functioned as an independent prognostic indicator using Cox regression analysis. Building upon the Cox results, we developed a relevant nomogram. The calibration curves demonstrated robust stability, and the timeROC curves assessed the precision of our model in forecasting the OS rates at 3-, 5-, and 10- year.

The five genes in our lysosome-related prognostic model have indicated a strong association with cancer. One of these genes, ATP6AP1, encodes the ATPase H+ transporting accessory protein 1. Serving as an essential subunit of the V-ATPase complex, it is essential for proton secretion and acidifying intracellular vesicles (34). Research by Tian et al. has shown that elevated ATP6AP1 expression in BC is inversely associated with survival, a finding consistent with our data (35). The amino acid transporter LAT1 (SLC7A5) has been recognized for its significance in cancer diagnostics and therapeutics, amplifying BC cell proliferation via the AKT/mTORC1 pathway and providing valuable prognostic information (36). A study revealed that EPDR1 had increased expression levels in bladder cancer tissues, which were associated with poorer survival results (37). Nevertheless, it was found to be a protective factor in BC, where EPDR1 inhibits malignant proliferation and encourages apoptosis in BC cells via the p53 signaling pathway (38). In gastric and colorectal cancers, the overexpression of SDC1 has been found to inhibit cancer cell growth. However, contrasting results were observed in pancreatic and breast tumors, where the overexpression of SDC1 had the opposite effect (39). Animal studies have confirmed SDC1’s role in facilitating BC brain metastasis (40). Meanwhile, the polymeric immunoglobulin receptor (PIGR), found on glandular epithelial cells, is positively associated with improved 5-year survival in BC patients when highly expressed (41). Instead of focusing solely on individual lysosomal genes in different cancer research, we merged them to construct risk models for BC prognosis, pointing towards a new direction for patient stratification.

Relevant studies have demonstrated the significance of lysosomes in not only being involved in the formation of the TIM but also aiding in the identification and stimulation of immune cells (42). The lysosomal activity of cells such as dendritic cells (DCs) and macrophages was found to correlate with the TIM (31). In our study, we observed significant correlations between the LRGs signature and effector memeory CD8 T cells as well as activated CD4 T cells. Our study revealed a negative correlation between the expression of ATP6AP1 and the infiltration of effector memeory CD8 T cells and B cells. The research conducted by Wang et al. exhibited similarities to our findings, indicating that ATP6AP1 might impact the prognosis of BC patients by affecting immune cell infiltration (43). Additionally, our study showed a statistically significant positive association between SLC7A5 and activated CD4 T cells. Previous research proposed that SLC7A5 acted in the activation of CD4+ T cells (44). Therefore, it was apparent that the LRGs signature substantially impacted the TIM in patients with BC. For this reason, we further explored whether there was a difference in TIM among BC patients who were categorized by their risk scores based on the LRGs signature. The findings showed that the low-risk group displayed higher levels of immune cells, specifically B cells, CD8 T cells, eosinophils, and mast cells. This suggested a favorable anti-tumor immune environment in the low-risk group. In contrast, the high-risk group had a diverse TIM with high concentrations of immune cells that promote tumor growth, such as Tregs and Th2, as well as immunological cells like natural killer (NK) cells and CD4/CD8 T cells, which halt tumor development. This intricate immune milieu suggested a potential link between aberrant immune infiltration and BC progression. Increased HLA Class I and II gene expression improves antigen presentation and supports T cell-mediated immune surveillance (45). Research has demonstrated that the poor processing and presentation of antigens by HLA-class I molecules contributes significantly to the growth of resistance to ICIs (46). Our findings indicated that the low-risk group demonstrated elevated expression of HLA genes, intimating an optimized immune surveillance milieu and potentially enhanced responsiveness to immunotherapy. In summary, our study indicated that the LRGs signature was closely linked to the TIM in BC, suggesting it could serve as a future target to modulate the immune microenvironment.

In the therapeutic landscape of BC, both immunotherapy and chemotherapy have garnered significant attention. Specifically, ICIs represent a crucial milestone in the evolution of oncological treatments (47). These agents enhance the T-cell immune response against tumors by modulating the activity of key molecules including CTLA-4, PD-1, and PD-L1. Recent research has shown the immune checkpoints are aberrant during degradation and presentation in lysosomes of cancer cells, suggesting they contribute to the mechanisms of tumor immune evasion (48). To delineate therapeutic responsiveness, we forecasted the efficacy of immunotherapy across two risk groups. Our evaluations, anchored in both TIDE and IPS methodologies, indicated a pronounced benefit of immunotherapy for low-risk BC patients. The GSE91061 cohort validated the capability of our signature to forecast the effectiveness of immunotherapy treatments. The GSE67501 cohort did not show a statistically significant difference in the proportion of patients with low-risk ratings who responded, but this could be attributed to constraints in the sample size of immunotherapy patients. Nonetheless, the findings from two immunotherapy cohorts hinted at a potential association between risk score and immunotherapy efficacy. Literature has denoted that treatments aimed at PD-1/PD-L1 are particularly potent in “hot tumors”, characterized by an abundant presence of CD8 T cells (49). Hence, synthesizing the insights from the immune microenvironment, IPS, and TIDE, it was inferred that tumors within the low-risk group predominantly align with the “hot tumor” profile, rendering them more amenable to subsequent immunotherapy. The results of our approach might offer valuable details for patients who have been sensitive to immunotherapy.

Furthermore, our risk scores exhibited a correlation with sensitivity to certain chemotherapeutic drugs that are common in clinical practice. Three chemotherapeutic medicines that exhibited a strong association with risk scores warrant our attention: Cytarabine, Olaparib, and Vinorelbine. Cytarabine, known for its role in inhibiting DNA synthesis *in vivo*, was predominantly employed in treating leukemias and lymphomas (50). The cellular lysosomal mass exhibited a notable increase subsequent to developing resistance to Cytarabine, as reported in a previous study (51). This observation suggested a potential involvement of lysosomes in the underlying mechanism of resistance to Cytarabin-based chemotherapy. Olaparib, an inhibitor of poly ADP-ribose polymerase (PARP), has received approval for the treatment of HER2-negative, germline BRCA1/2-mutated metastatic BC (52). Recent studies indicated that in prostate cancer cells, Olaparib undergoes lysosomal degradation, a pathway potentially linked to drug resistance (53). Vinorelbine, a periwinkle alkaloid derivative, has been a conventional therapeutic for metastatic BC (54). In summation, our data proposed a theoretical foundation for the role of lysosomes in influencing the chemotherapeutic drug sensitivity, thereby offering additional insight into BC treatment strategies.

In our investigation, the lysosomal prognostic model demonstrated notable predictive efficacy across a range of BC datasets. Significantly, enhanced by the integration of multiple LRGs, our model displayed good accuracy in forecasting long-term survival outcomes for BC patients. Yet, we recognize the limitations of our study. Although clinical experiments were conducted to validate the study, only a small number of patient specimens were collected, and we will continue collecting more samples for further research. Furthermore, while extensive datasets from public databases lend clinical significance to our findings, integrating more detailed clinical data could further sharpen our prognostic accuracy. Our prognostic model also has to be tested in multicenter cohorts to see how well it performs.

## 5 Conclusion

We were successful in developing and validating a unique LRGs signature to predict survival in BC. The present work elucidated LRGs had significant correlations with prognosis of BC, immune status, and drug sensitivity. It is anticipated that these genes possess the ability to serve as prognostic indicators and exert an impact on the efficacy of immunotherapy for BC.

## Data availability statement

The original contributions presented in the study are included in the article/**Supplementary Material**. Further inquiries can be directed to the corresponding author.

## Ethics statement

The studies involving humans were approved by the Ethics Committee of the Second Affiliated Hospital of Guangzhou University of Chinese Medicine. The studies were conducted in accordance with the local legislation and institutional requirements. The participants provided their written informed consent to participate in this study.

## Author contributions

HS: Conceptualization, Data curation, Writing – original draft, Writing – review & editing, Validation. YC: Conceptualization, Data curation, Validation, Writing – original draft, Writing – review & editing. FL: Formal Analysis, Writing – review & editing. WL: Formal Analysis, Writing – review & editing. XG: Formal Analysis, Writing – review & editing. WZ: Resources, Writing – review & editing. DL: Resources, Writing – review & editing. ML: Writing – review & editing. SZ: Writing – review & editing. QC: Writing – review & editing. QC: Project administration, Supervision, Writing – review & editing.
