# Supplementary figures and images for: Establishment of a lysosome-related prognostic signature in breast cancer to predict immune infiltration and therapy response
Source: Front Oncol. 2023 Dec 14;13:1325452. doi: 10.3389/fonc.2023.1325452 (PMC10757638; doi:10.3389/fonc.2023.1325452)

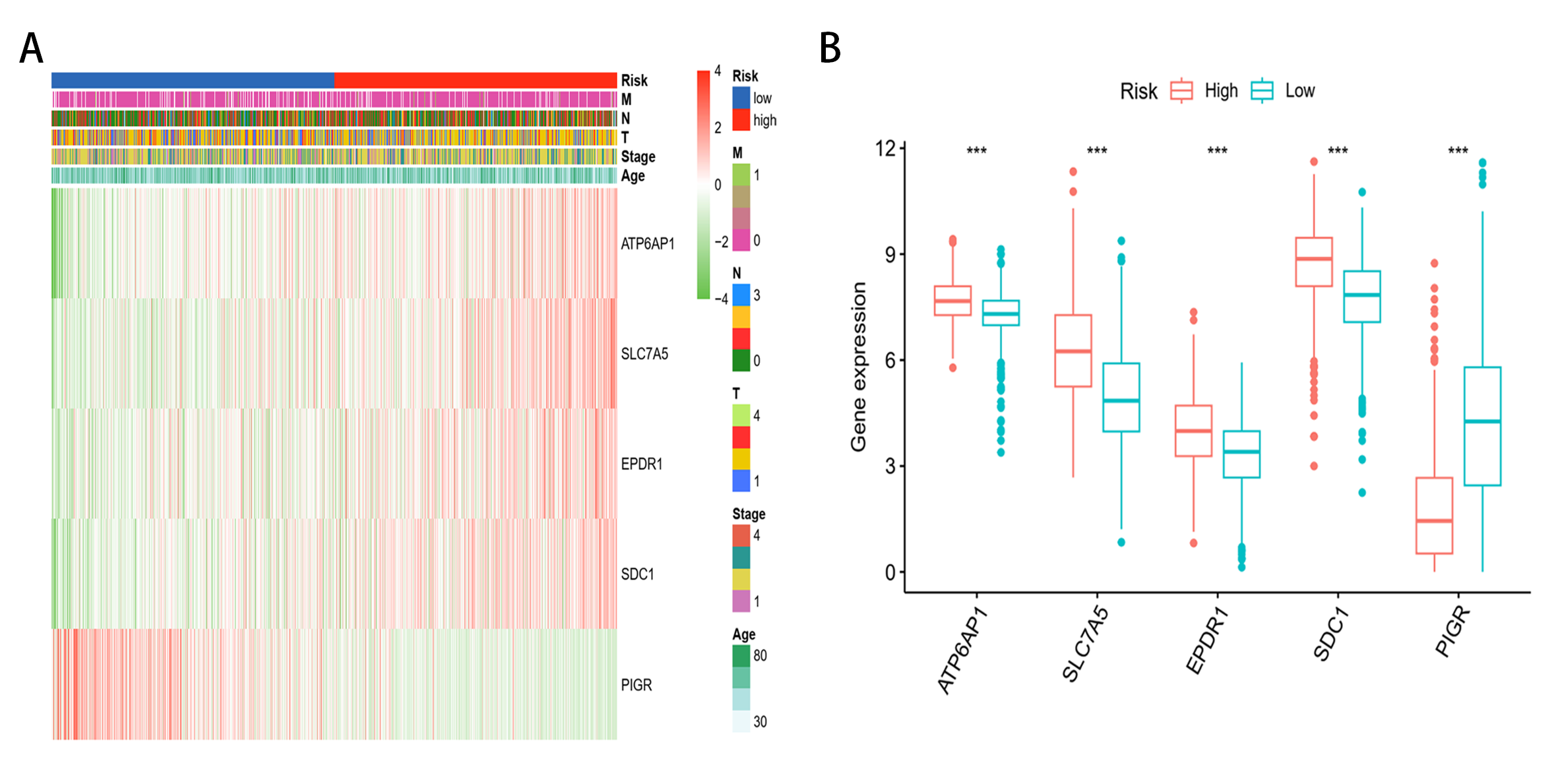

Supplement: Supplementary file 1 [file Image_1.tif]

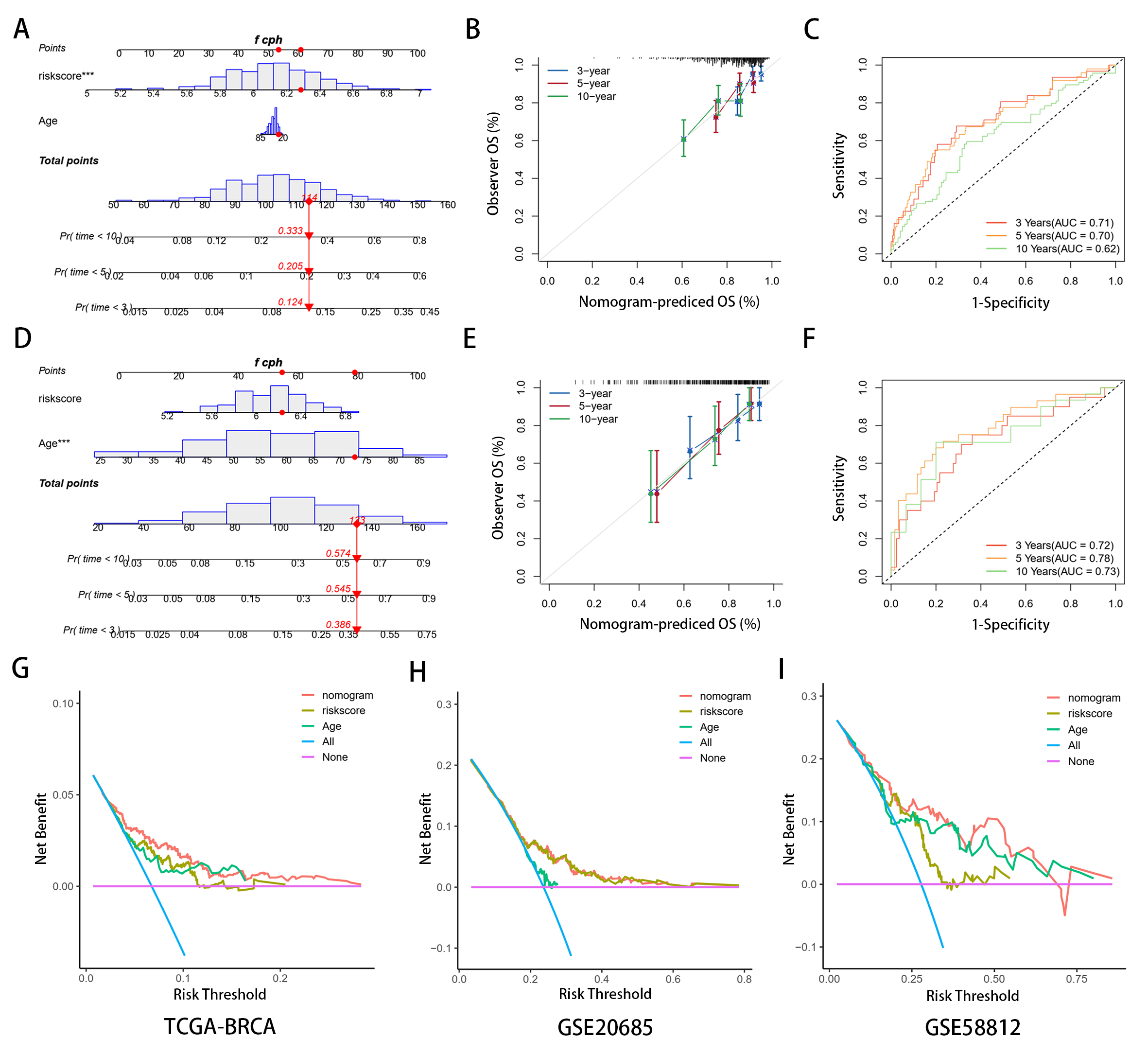

Supplement: Supplementary file 2 [file Image_2.tif]

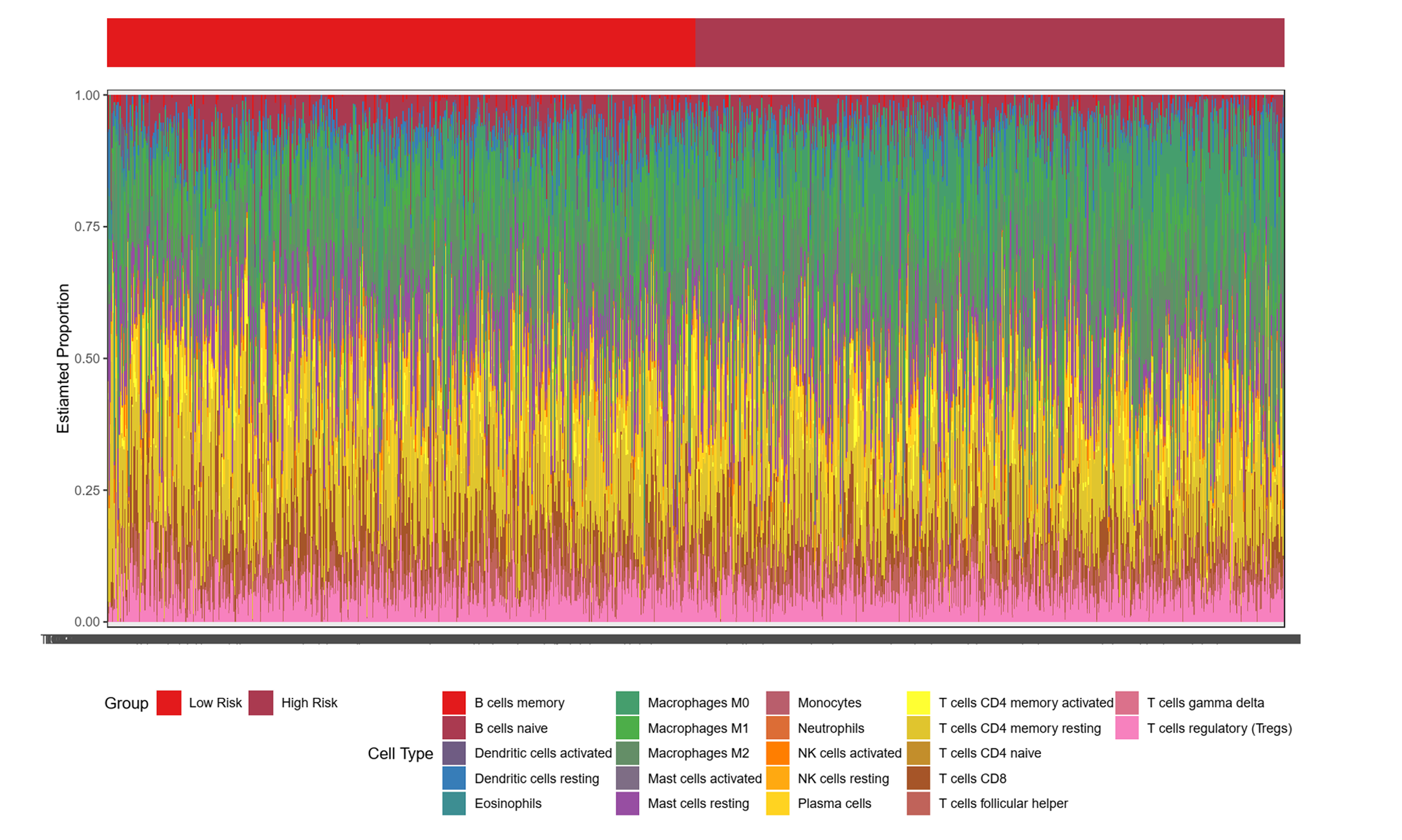

Supplement: Supplementary file 3 [file Image_3.tif]

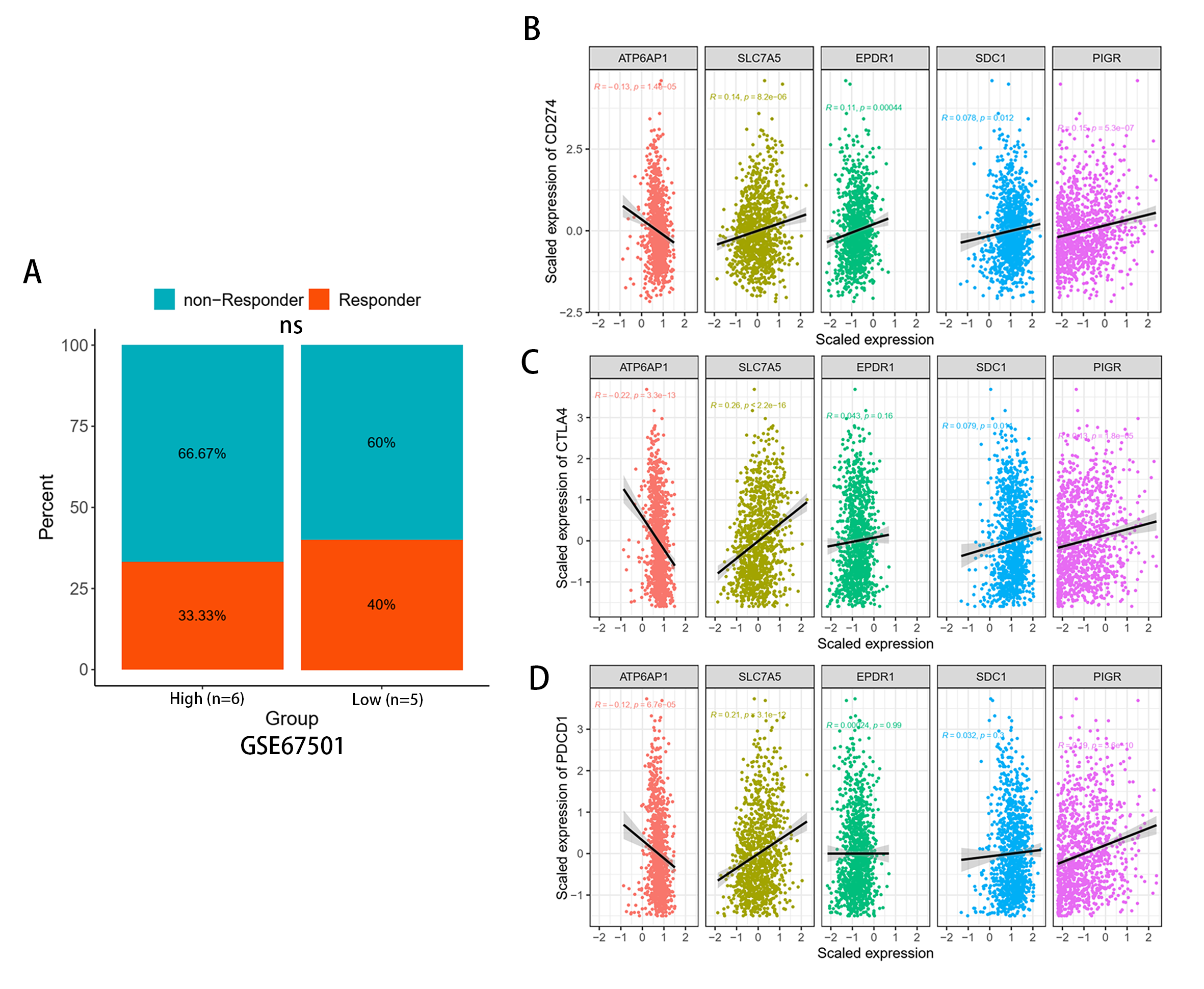

Supplement: Supplementary file 4 [file Image_4.tif]

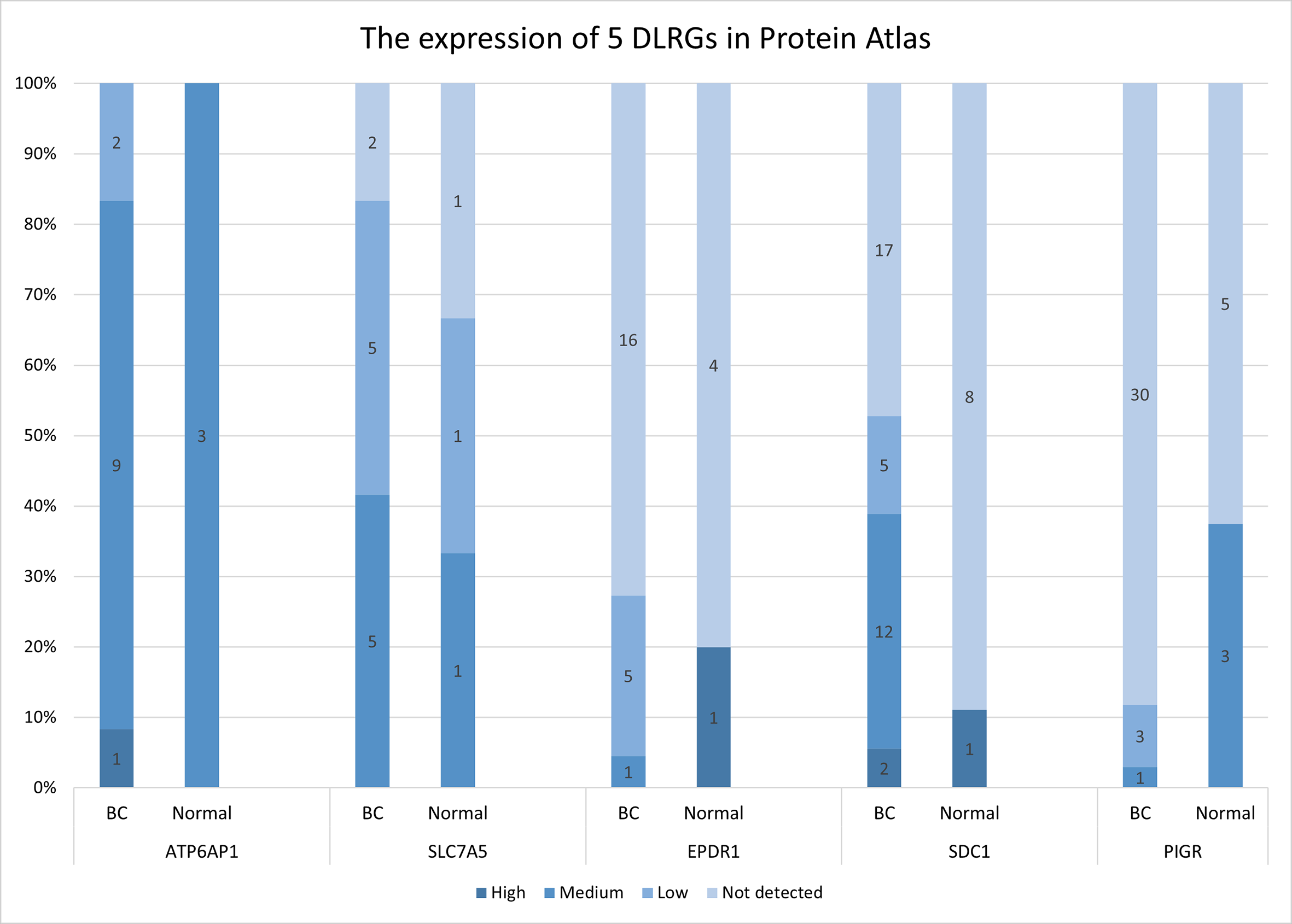

Supplement: Supplementary file 5 [file Image_5.tif]
