# Supplementary material for: Establishment of a lysosome-related prognostic signature in breast cancer to predict immune infiltration and therapy response
Source: Front Oncol. 2023 Dec 14;13:1325452. doi: 10.3389/fonc.2023.1325452 (PMC10757638; doi:10.3389/fonc.2023.1325452)
Supplement: Supplementary file 6 [file Table_1.docx]

**Supplementary Table 1. The clinical characteristics of breast cancer samples in the TCGA training set, GSE20685, and GSE58812 validation sets.**

| **Characteristics** | **TCGA cohort** | **GSE20685 cohort** | **GSE58812 cohort** |
| --- | --- | --- | --- |
| **n** | 1049 | 327 | 107 |
| **Age, n (%)** |  |  |  |
| ≤65 | 756 (72.1%) | 305 (93.3%) | 72 (67.3%) |
| ＞65 | 293 (27.9%) | 22 (6.7%) | 35 (32.7%) |
| **WHO stage, n (%)** |  |  |  |
| Ⅰ | 183 (17.5%) | / | / |
| Ⅱ | 592 (56.4%) |  |  |
| Ⅲ | 233 (22.2%) |  |  |
| Ⅳ | 18 (1.7%) |  |  |
| Ⅹ | 12 (1.1%) |  |  |
| Unknown | 11 (1.1%) |  |  |
| **T stage, n (%)** |  |  |  |
| T1 | 280 (26.7%) | 101 (30.9%) | / |
| T2 | 599 (57.1%) | 188 (57.5%) |  |
| T3 | 132 (12.6%) | 26 (7.9%) |  |
| T4 | 35 (3.3%) | 12 (3.7%) |  |
| TX | 3 (0.3%) |  |  |
| **N stage, n (%)** |  |  |  |
| N0 | 491 (46.8%) | 137 (41.9%) | / |
| N1 | 356 (34.0%) | 87 (26.6%) |  |
| N2 | 110 (10.5%) | 63 (19.3%) |  |
| N3 | 75 (7.1%) | 40 (12.2%) |  |
| NX | 17 (1.6%) |  |  |
| **M stage, n (%)** |  |  |  |
| M0 | 867 (82.7%) | 319 (97.6%) | / |
| M1 | 20 (1.9%) | 8 (2.4%) |  |
| MX | 162 (15.4%) |  |  |
| **Survival status, n (%)** |  |  |  |
| Alive | 898 (85.6%) | 244 (74.6%) | 78 (72.9%) |
| Dead | 151 (14.4%) | 83 (25.4%) | 29 (27.1%) |
